# Sarcopenia Predicts Outcome After Chemoimmunotherapy, Not Chemotherapy, in Advanced Lung Cancer: Single‐Centre Retrospective Study

**DOI:** 10.1002/jcsm.70243

**Published:** 2026-03-25

**Authors:** Hyojin Lee, Chang Gon Kim, Sookyeong Han, Su Kyoung Park, Hong In Yoon, Kyung Hwan Kim, Hyo Sup Shim, Min Hee Hong, Ja Hyun Yeo, Sangwoo Kim, Sang Hyun Hwang, Hye Ryun Kim, Namki Hong

**Affiliations:** ^1^ Division of Medical Oncology, Department of Internal Medicine, Yonsei Cancer Center Yonsei University College of Medicine Seoul Republic of Korea; ^2^ Department of Internal Medicine, Endocrine Research Institute Yonsei University College of Medicine Seoul Republic of Korea; ^3^ Institute for Innovation in Digital Healthcare (IIDH) Yonsei University Health System Seoul Republic of Korea; ^4^ Department of Medical Records, Severance Hospital Yonsei University College of Medicine Seoul Republic of Korea; ^5^ Department of Radiation Oncology, Yonsei Cancer Center Yonsei University College of Medicine Seoul Republic of Korea; ^6^ Department of Pathology Yonsei University College of Medicine Seoul Republic of Korea; ^7^ Department of Biomedical Systems Informatics and Brain Korea 21 PLUS Project for Medical Science Yonsei University College of Medicine Seoul Republic of Korea; ^8^ Department of Nuclear Medicine, Severance Hospital Yonsei University College of Medicine Seoul Republic of Korea

**Keywords:** advanced non‐small cell lung cancer, chemoimmunotherapy, chemotherapy, predictive marker, sarcopenia, skeletal muscle analysis

## Abstract

**Background:**

Sarcopenia, characterized by progressive loss of skeletal muscle mass, is prevalent in patients with non‐small cell lung cancer (NSCLC). While sarcopenia has been associated with poor prognosis in multiple types of cancer, its predictive role in the context of first‐line treatment combining PD‐(L)1 inhibitors with platinum‐based chemotherapy (CITx) remains unclear in advanced NSCLC.

**Methods:**

In a single‐centre retrospective cohort, patients with advanced NSCLC without actionable genomic alterations who received either CITx (*n* = 552) or platinum‐doublet chemotherapy alone (CTx; *n* = 622) were analysed. Sarcopenia was defined on the Martin's computed tomography‐derived skeletal muscle index definition. Progression‐free survival (PFS) and overall survival (OS) were assessed. Interaction between sarcopenia and treatment modality was explored.

**Results:**

Sarcopenia was observed in 49.5% of patients. The presence of sarcopenia was associated with worse outcomes in overall patients (median OS: 9.4 vs. 11.4 months; HR 1.22, 95% CI 1.08–1.39). When stratified by treatment groups, sarcopenia was significantly associated with higher risk of progression (adjusted HR 1.23, 95% CI 1.01–1.49) and death (adjusted HR 1.42, 95% CI 1.16–1.74) in CITx‐treated patients, whereas no such association was observed in the CTx group. The interaction between sarcopenia and treatment type was significant for both PFS (*p* = 0.041) and OS (*p* = 0.022).

**Conclusions:**

Sarcopenia is a predictive biomarker for inferior outcomes in patients with advanced NSCLC treated with CITx, but not with CTx. Assessment of skeletal muscle mass can help identify patients at risk for suboptimal response to CITx. This study provides grounds for future exploration in interventions targeting sarcopenia and cachexia to enhance clinical outcomes in advanced cancer patients.

## 1 Introduction

Sarcopenia is a medical condition characterized by the progressive loss of skeletal muscle mass, strength and function [1]. Sarcopenia is prevalent among patients with chronic disease including multiple types of cancer [2], consistently correlating with lower survival in patients with cancer [1, 3]. It is well known that sarcopenia can lead to higher functional disability and mortality in patients receiving systemic treatment for advanced cancer [4], which highlights the clinical relevance of musculoskeletal integrity as it may dictate treatment outcomes. Moreover, recent studies suggest that muscle wasting has been implicated in shaping unfavourable tumour microenvironment and hinders systemic immune response [5]. Correspondingly, depletion of muscle mass was associated with worse efficacy and increased toxicity of checkpoint blockade in cancer types including melanoma, hepatocellular carcinoma and breast cancer [6].

Sarcopenia is commonly observed in patients with non‐small cell lung cancer (NSCLC) [7], the leading cause of cancer‐related deaths globally [8]. In the past decades, platinum‐based chemotherapy has been the standard of treatment for patients with NSCLC [9]. Recently, the advent of immune checkpoint blockade targeting programmed cell death‐1 (PD‐1) or its ligand PD‐L1 has revolutionized the treatment landscape for advanced NSCLC [10, 11]. PD‐(L)1 blockade is preferred over chemotherapy alone in advanced NSCLC without actionable genomic alterations, either in combination with chemotherapy or as monotherapy depending on PD‐(L)1 expression [12]. Nonetheless, a predictive biomarker for the treatment outcome of PD‐(L)1 blockade plus platinum‐based chemotherapy is still limited [13], and important factors such as sarcopenia status are not currently integrated into treatment decision‐making.

Studies have shown that loss of skeletal muscle mass was associated with poor outcomes in patients with NSCLC treated with PD‐(L)1 blockade [14, 15, 16]. However, no studies have addressed whether sarcopenia serves as a predictive biomarker for differential benefit from chemoimmunotherapy versus chemotherapy in patients with advanced NSCLC. Therefore, we aimed to investigate whether body composition analysis focusing on muscle mass can specifically predict the treatment outcome of first‐line systemic treatment with PD‐(L)1 inhibitor plus platinum‐doublet compared to platinum‐based chemotherapy alone in a single‐centre cohort of patients with advanced NSCLC.

## 2 Methods

### 2.1 Patients

**FIGURE 1:**
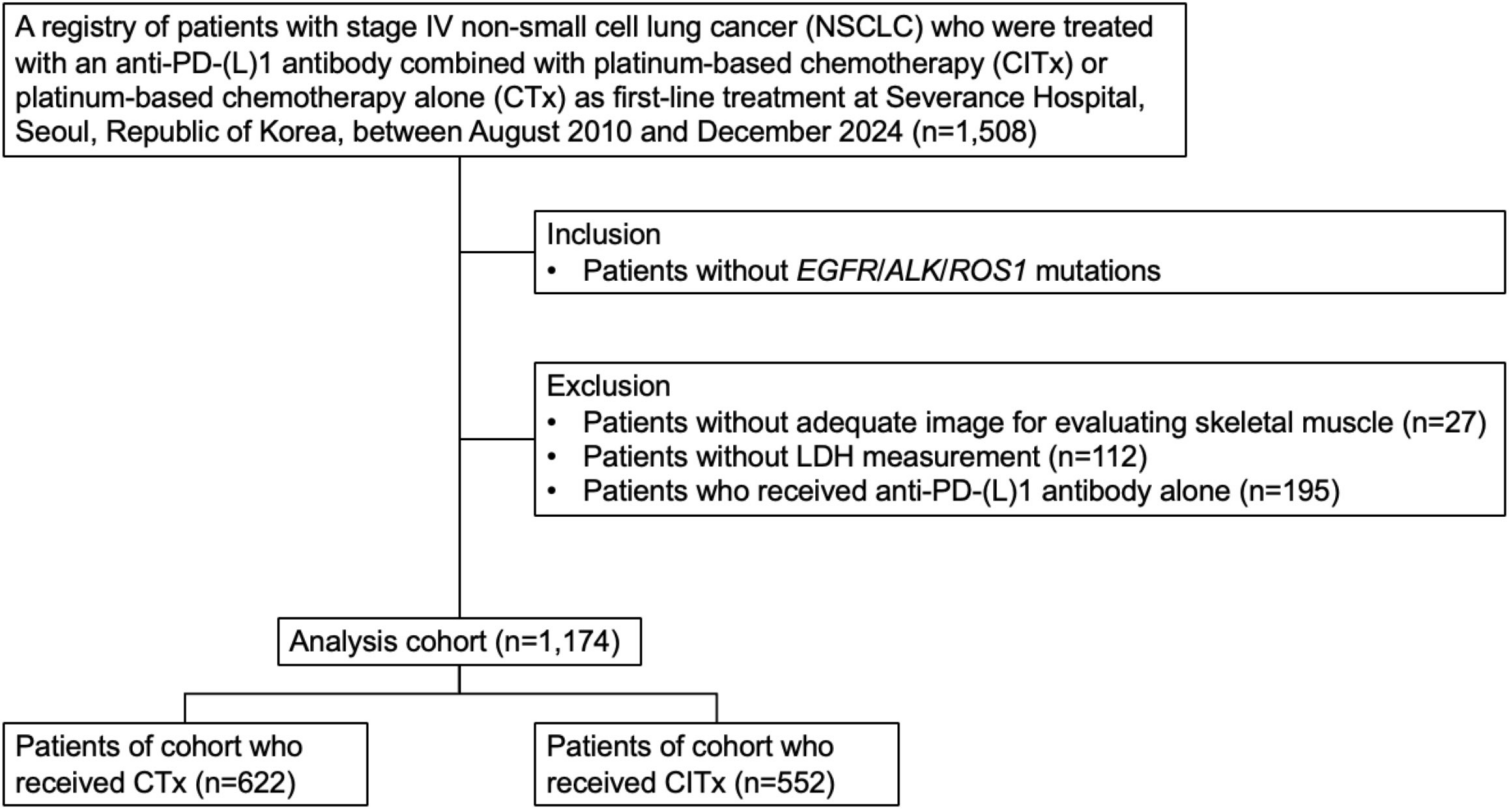
Flow diagram of patient inclusion and exclusion criteria. LDH, lactate dehydrogenase; PD‐(L)1, programmed cell death protein 1 (PD‐1) or its ligand (PD‐L1).

We retrospectively analysed 1508 patients with Stage IV metastatic NSCLC without sensitizing *EGFR*, *ALK* or *ROS1* who had received no previous treatment for advanced disease between August 2010 and December 2024 in Severance Hospital, South Korea. Patients were excluded if they lacked adequate imaging for skeletal muscle evaluation (*N* = 27), if baseline serum lactate dehydrogenase (LDH) was unavailable (*N* = 112) or if they received anti‐PD‐(L)1 antibody alone (*N* = 195). A total of 1174 patients were included in the final analysis, including patients who were treated with either an anti‐PD‐(L)1 antibody combined with platinum‐based chemotherapy (CITx; *N* = 552) or platinum‐based chemotherapy (CTx; *N* = 622) as a first‐line treatment (Figure 1). Patients receiving immunotherapy alone were excluded as this modality of treatment has not been covered by National Health Insurance as a first‐line treatment yet in Republic of Korea. Treatment continued until radiographic progression, unacceptable toxicity, a decision by the treating physician to discontinue treatment, or patient refusal of further treatment. Data on the following variables were collected: age, sex, height, weight, enrollment era, the Eastern Cooperative Oncology Group (ECOG) performance status, PD‐L1 expression measured as a tumour proportion score, smoking history, LDH level, albumin level, neutrophil‐to‐lymphocyte ratio (NLR), metastatic sites and tumour histology. Lung immune prognostic index (LIPI) score was calculated based on categories of LDH level (cutoff: upper limit of LDH) and derived NLR (dNLR, absolute neutrophil count/[white blood cell count minus absolute neutrophil count]; cutoff: 3) [17]. The Institutional Review Board of Yonsei University College of Medicine approved this study (IRB approved no. 4‐2023‐0034).

### 2.2 Image Protocols and Body Composition Analysis

Baseline non‐contrast CT images of study patients were obtained from ^18^F‐fluorodeoxyglucose positron emission tomography/computed tomography (PET/CT) to evaluate distant metastasis. The dataset included images acquired up to 90 days (median difference between image acquisition date and treatment initiation date: 16 days [interquartile range 9–27 days]) before the start of first‐line systemic treatment. A dedicated commercialized deep learning software (Deepcatch, v1.2.0.6619, MEDICALIP Co. Ltd., Seoul, Korea) was used to obtain body composition indices from the CT scans [18]. This software allows the semiautomatic segmentation and quantification of different body composition components including abdominal visceral fat area (VFA), subcutaneous fat area (SFA) and skeletal muscle area (SMA; Figure S1). The software utilizes a deep neural network algorithm to segment and quantify bone, muscle and fat in CT axial images, showing good accuracy (dice similarity coefficient for skeletal muscle and fat region: 0.97–0.99 compared with a standard reference generated by a human expert) and reproducibility (intraclass correlation using a two‐way random effects model: 0.99; 95% confidence interval [CI]: 0.98–0.99) [18]. After the automated segmentation of L3 cross‐sectional images, manual inspection and correction for any potential errors in the segmentation process were performed to ensure segmentation quality. All images in this study were analysed by a single operator with more than 8 years of experience in image analysis.

### 2.3 Sarcopenia Definitions

Skeletal muscle index (SMI) was calculated as SMA / height (m)^2^. Sarcopenia was defined using Martin's definition using CT‐derived SMI (SMI < 43 cm^2^/m^2^ for men with body mass index [BMI] < 25 kg/m^2^; SMI < 53 cm^2^/m^2^ for men with BMI ≥ 25 kg/m^2^; SMI < 41 cm^2^/m^2^ for women) in main analyses [3]. For sensitivity analyses to test the robustness of findings, two additional sarcopenia definitions based on CT‐derived SMI were used (median sarcopenia: < sex‐stratified median of SMI in the study population [< 45.6 cm^2^/m^2^ for men and < 39.4 cm^2^/m^2^ for women]; sarcopenia defined by Korean‐specific threshold: two standard deviations below the average SMI value in a healthy Korean population; < 40.96 cm^2^/m^2^ for men and < 30.60 cm^2^/m^2^ for women) [19]. In addition, standardized SMI (sex‐stratified) was included in the statistical models to test the association between muscle mass as a continuous variable and outcomes.

### 2.4 Follow‐Up and Response Evaluation

Laboratory tests and physical examinations were performed at every cycle of administration with a systemic treatment. Treatment response was evaluated via imaging analysis, including CT and magnetic resonance imaging. Tumour imaging was performed at Weeks 6 and 12, every 9 weeks until Week 48 and every 12 weeks after then. Based on Response Evaluation Criteria in Solid Tumors (RECIST) Version 1.1, treatment response was classified as complete response, partial response, stable disease or progressive disease [20]. The data cut‐off date was 31 December 2024. During the study period, 34 patients were lost during follow‐up and were censored at the last date of known survival status.

### 2.5 Statistical Analysis

Clinical characteristics of study patients with or without sarcopenia were compared using two‐sample independent *t*‐test, Wilcoxon rank‐sum test and chi‐squared test as appropriate. PFS was measured as the time from the initiation of treatment to disease progression or death. Overall survival (OS) was measured as the time from the initiation of treatment to death from any cause. Survival curves were plotted using the Kaplan–Meier method, and differences between subgroups were compared using the log‐rank test. Cox proportional hazards model was used for the univariate and multivariable analyses. Variables included in the multivariable Cox proportional hazard model: sarcopenia, treatment groups (CITx vs. CTx), enrollment era (defined according to whether patients were enrolled before or after the introduction of reimbursement by national health insurance), age, sex, BMI, smoking history (pack‐years), NLR, LIPI score, pathology type, performance status and PD‐L1 expression. To evaluate potential effect modification of the association between sarcopenia and outcomes by treatment group and enrollment era, we fitted a multivariable model including main effects for sarcopenia, treatment group, and enrollment era, covariates, all lower‐order interaction terms and a three‐way interaction term (sarcopenia × treatment group × enrollment era). No violation of proportional hazard assumption was observed. Interaction between the presence of sarcopenia and treatment groups (CITx and CTx) was tested in multivariable Cox proportional hazard models. All statistical analyses were performed using R Version 4.0.4 (http://www.R‐project.org), GraphPad Prism Version 6.0 (GraphPad Software, San Diego, CA) and Stata 18.0 (Statacorp, TX). Two‐sided *p*‐values < 0.05 were considered significant.

## 3 Results

### 3.1 Patient Characteristics

This study included 1174 patients, including 581 with sarcopenia and 593 without sarcopenia (Table 1). The median age was 65.8 years (range: 25–90 years), with 935 males (79.6%) and 239 females (20.4%). Most patients were current (40.1%) or former smokers (38.4%). Brain metastases were observed in 409 patients (34.8%), while 148 (12.6%) had liver metastases. Patients with sarcopenia (*n* = 581, 49.5%) were older, had lower BMI, higher NLR, a higher proportion of poor LIPI score and lower VFA and SFA. Performance status and PD‐L1 expression did not differ between patients with and without sarcopenia. The proportion of patients treated with CITx did not differ significantly between the sarcopenia and non‐sarcopenia groups (47.5% vs. 46.5%; *p* = 0.741). Both grade ≥ 3 toxicities (76.4% vs. 71.8%, *p* = 0.073; CTCAE v5.0) and treatment discontinuation due to toxicity (9.5% vs. 6.5%, *p* = 0.052) were numerically higher in patients with sarcopenia compared with those without sarcopenia, but neither difference reached statistical significance.

**TABLE 1:** Characteristics of patients with Stage IV metastatic NSCLC.

Characteristics	Overall (*n* = 1174)	With sarcopenia (*n* = 581, 49.5%)	Without sarcopenia (*n* = 593, 50.5%)	*p*
Age, year	65.8 ± 10.2	67.3 ± 10.1	64.3 ± 10.1	< 0.001
Men, *n* (%)	935 (79.6)	431 (74.2)	504 (85.0)	< 0.001
Body mass index, kg/m^2^	23.0 ± 3.1	22.1 ± 3.2	23.8 ± 2.8	< 0.001
ECOG PS ≥ 2	148 (12.6)	74 (12.7)	74 (12.5)	0.894
Smoking history, pack‐years	30.0 [8.5–45.0]	30.0 [0.0–45.0]	30.0 [14.0–41.0]	0.516
Enrollment era (post‐reimbursement)	288 (24.5)	151 (26.0)	137 (23.1)	0.250
Neutrophil‐to‐lymphocyte ratio	3.6 [2.4–5.7]	3.9 [2.5–6.1]	3.4 [2.4–5.4]	0.005
Lactate dehydrogenase	226 [187–304]	224 [185–305]	228 [189–297]	0.361
Lung immune prognostic index score				0.013
0	528 (45.0)	264 (45.4)	264 (44.5)	
1	487 (41.5)	223 (38.4)	264 (44.5)	
2	159 (13.5)	94 (16.2)	65 (11.0)	
Higher pathology types				0.716
Squamous, *n* (%)	331 (28.2)	161 (27.7)	170 (28.7)	
Non‐squamous, *n* (%)	843 (71.8)	420 (72.3)	423 (71.3)	
PD‐L1 expression, *n* (%)				0.077
0%	474 (40.4)	219 (37.7)	255 (43.0)	
1%–49%	366 (31.2)	201 (34.6)	165 (27.8)	
≥ 50%	211 (18.0)	104 (17.9)	107 (18.0)	
Unknown	123 (10.4)	57 (9.8)	66 (11.2)	
Treatment regimen, *n* (%)				0.741
Chemotherapy	622 (53.0)	305 (52.5)	317 (53.5)	
Immunotherapy plus chemotherapy	552 (47.0)	276 (47.5)	276 (46.5)	
Chemotherapy dose reduction, %	92.8 ± 12.5	91.4 ± 13.3	94.2 ± 11.5	< 0.001
Radiotherapy, *n* (%)	167 (14.2)	88 (15.2)	79 (13.3)	0.371
L3 CT body compositions				
SMA, cm^2^	122.1 ± 23.9	108.5 ± 19.7	135.4 ± 20.1	< 0.001
SMI, cm^2^/m^2^	44.7 ± 7.5	39.7 ± 5.5	49.6 ± 5.7	< 0.001
SFA, cm^2^	114.4 ± 55.1	110.4 ± 57.1	118.3 ± 52.9	0.014
SFI, cm^2^/m^2^	42.7 ± 22.4	41.2 ± 22.6	44.1 ± 22.1	0.027
VFA, cm^2^	113.9 ± 67.3	106.8 ± 71.7	120.8 ± 61.9	< 0.001
VFI, cm^2^/m^2^	41.8 ± 24.5	39.0 ± 25.5	44.5 ± 23.2	< 0.001

*Note:* Data were presented as mean ± standard deviation, median [interquartile range] or number (%). Sarcopenia was defined using Martin's definition (J Clin Oncol. 2013; 31:1539–1547. doi:10.1200/JCO.2012.45.2722).

Abbreviations: ECOG PS, Eastern Cooperative Oncology Group Performance Status; L3, third lumbar spine level; SFA, subcutaneous fat area; SFI, subcutaneous fat index; SMA, skeletal muscle area; SMI, skeletal muscle index; VFA, visceral fat area; VFI, visceral fat index.

### 3.2 Survival Outcomes According to Treatment Groups

Detailed treatment regimens are presented in Table S1. During median follow‐up (event‐free) period of 46.7 months (95% CI 42.9–53.0), deaths and progression were reported in 985 (83.9%) patients and 1107 (94.3%) patients, respectively. The median PFS and OS were 5.4 months (95% CI 4.8–6.1) and 11.1 months (95% CI 9.6–12.5) in the CITx, and 3.9 months (3.5–4.3) and 9.5 months (95% CI 8.2–10.7) in the CTx group, respectively (Figure S2A,B). PFS and OS at 12 months were higher in the CITx group (PFS 27%, 95% CI 23–30; OS 47%, 95% CI 43–51) compared to the CTx group (PFS 14.4%, 95% CI 12–17; OS 41%, 95% CI 37–45). Hazard ratio (HR) of CITx (reference: CTx) for progression of disease and for death was 0.69 (95% CI 0.61–0.77) and 0.87 (95% CI 0.76–0.98), respectively (Table S2).

### 3.3 Survival Outcomes According to Sarcopenia

**FIGURE 2:**
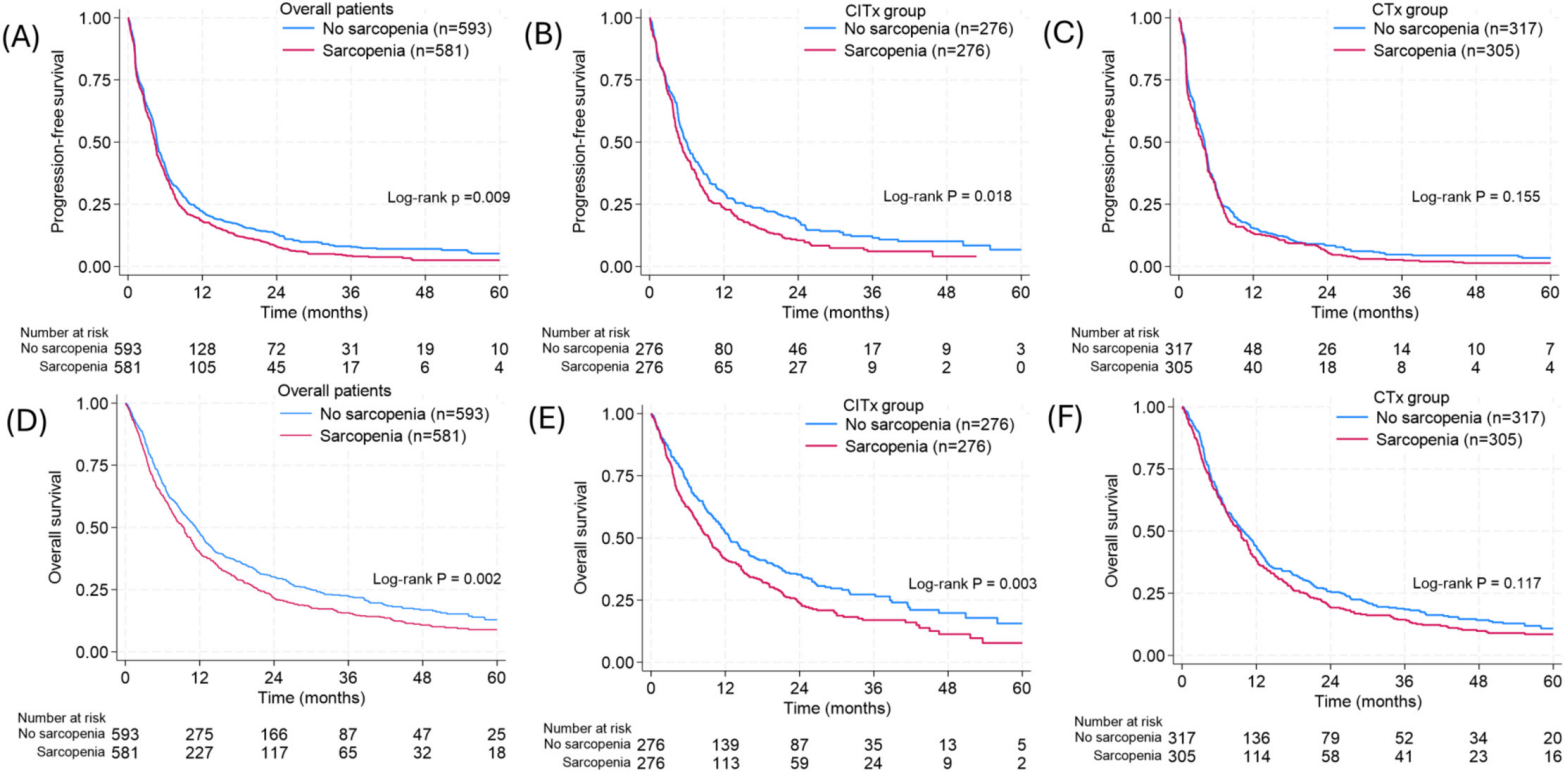
PFS and OS in patients receiving chemotherapy or chemoimmunotherapy grouped by presence of sarcopenia. (A) PFS and (D) OS in overall patients. (B) PFS and (E) OS in patients receiving CITx. (C) PFS and (F) OS in patients receiving CTx. CITx, chemoimmunotherapy; CTx, chemotherapy; OS, overall survival; PFS, progression‐free survival.

When patients were grouped by the presence of sarcopenia, the median PFS and OS in the sarcopenia group was lower (PFS 4.3 months, 95% CI 3.9–4.6; OS 9.4 months, 95% CI 8.0–10.4) compared to the no sarcopenia group (PFS 4.7 months, 95% CI 4.5–5.0; OS 11.4 months, 95% CI 9.9–12.6; Figure 2A,D). Patients with sarcopenia had a higher risk of progression (HR 1.17, 95% CI 1.04–1.31) and death (HR 1.22, 95% CI 1.08–1.39) compared to those without (Table S2).

### 3.4 Interaction Between Sarcopenia and Treatment Groups

**FIGURE 3:**
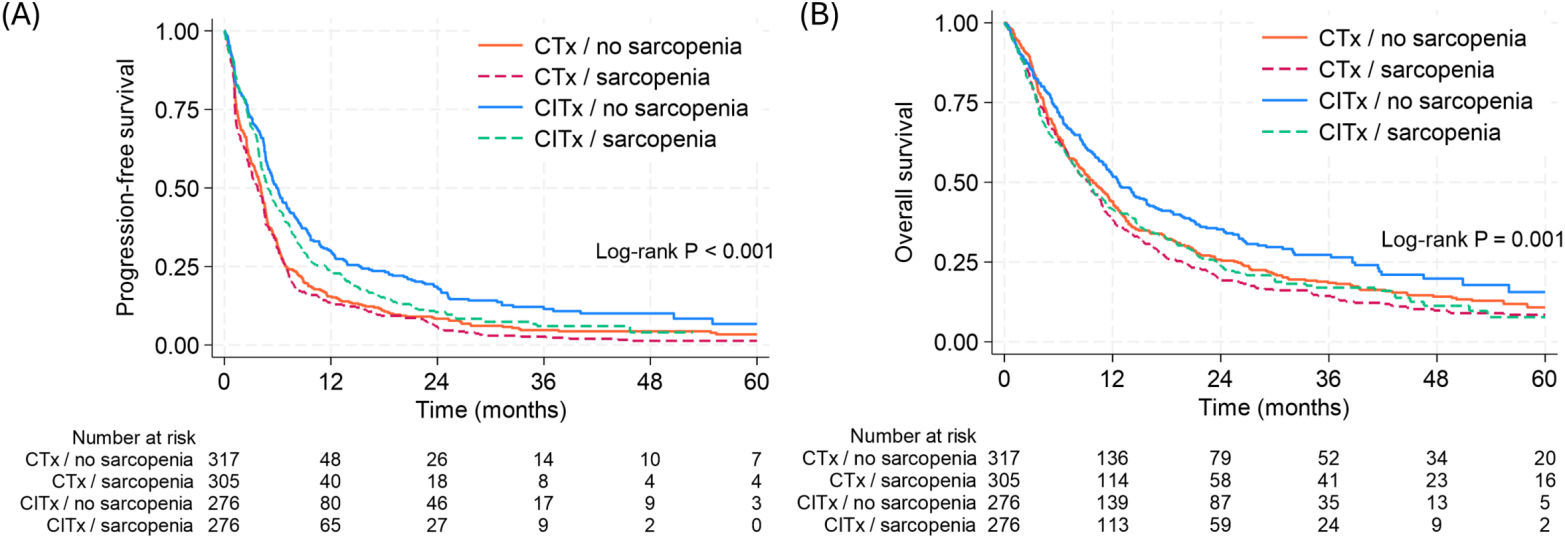
Survival rates by combination of treatment regimen and presence of sarcopenia. (A) PFS and (B) OS in patients receiving CTx without sarcopenia, CTx with sarcopenia, CITx without sarcopenia and CITx with sarcopenia. CITx, chemoimmunotherapy; CTx, chemotherapy; OS, overall survival; PFS, progression‐free survival.

When patients were stratified by sarcopenia status within treatment groups (CITx and CTx), absence of sarcopenia was associated with better PFS and OS in CITx group (Figure 2B,E). However, in CTx group, PFS and OS did not differ by presence of sarcopenia (Figure 2C,F). In CITx group, patients with sarcopenia had greater risk of progression (adjusted HR [aHR] 1.23, 95% CI 1.01–1.49) or death (aHR 1.42, 95% CI 1.16–1.74) after adjustment for age, sex, enrollment era, BMI, smoking history, NLR, LIPI score, pathology types, performance status and PD‐L1 expression than those without, whereas the presence of sarcopenia was not associated with PFS or OS in CTx group. The effect modification by treatment groups for the association between sarcopenia and outcomes was statistically significant (*p* for interaction 0.041 and 0.022 for PFS and OS, respectively; Table 2). Among the interaction terms, only the two‐way interaction between sarcopenia and treatment group was statistically significant, whereas interactions involving enrollment era, including the three‐way interaction (sarcopenia × treatment group × enrollment era), were not statistically significant. These findings indicate that the effect modification of treatment by sarcopenia was consistent across enrollment eras. In sensitivity analyses using Korean‐specific threshold or median threshold to define sarcopenia (Table S3; Figure S3), presence of sarcopenia remained as an independent predictor of treatment outcomes only in CITx group but not in CTx group when different definitions of sarcopenia were applied. In analysis using SMI as continuous variable, one standard deviation increment in SMI (sex‐stratified) was associated with 11% (aHR 0.89, *p* = 0.049) and 17% (aHR 0.83, *p* = 0.002) lower hazard of progression and death, independent of visceral fat index, subcutaneous fat index and other covariates in CITx group but not in CTx group (Table S4). When patients were grouped into four groups by combination of treatment and the presence of sarcopenia (Figure 3A,B), patients without sarcopenia who were treated with CITx had the lowest risk of progression and death (aHR 0.54 and 0.70), followed by CITx with sarcopenia (aHR 0.67 and 0.96), CTx with no sarcopenia (aHR 0.97 and 0.96) and CTx with sarcopenia (referent) groups (Table S5).

**TABLE 2:** Hazard ratios of sarcopenia for progression‐free survival and overall survival by treatment groups.

Outcomes	Treatment groups	Predictor	Univariate model		Multivariable model^a^		
			Unadjusted HR (95% CI)	*p*	Adjusted HR (95% CI)	*p*	*p* for interaction^b^ (sarcopenia × treatment group)
PFS	CITx	Sarcopenia	1.24 (1.04–1.48)	0.018	1.23 (1.01–1.49)	0.036	0.041
	CTx		1.12 (0.95–1.31)	0.155	1.04 (0.87–1.25)	0.648	
OS	CITx	Sarcopenia	1.33 (1.10–1.61)	0.003	1.42 (1.16–1.74)	0.001	0.022
	CTx		1.14 (0.96–1.34)	0.118	0.99 (0.82–1.19)	0.985	

Abbreviations: CI, confidence interval; HR, hazard ratio; OS, overall survival; PFS, progression‐free survival; ref, reference group.

^a^ Variables included in the multivariable Cox proportional hazard model: sarcopenia, treatment groups (CITx vs. CTx), enrollment era (defined according to whether patients were enrolled before or after the introduction of reimbursement by national health insurance), age, sex, body mass index, smoking history (pack‐years), neutrophil‐to‐lymphocyte ratio, lung immune prognostic index (LIPI) score, pathology type, performance status and PD‐L1 expression.

^b^ To evaluate potential effect modification of the association between sarcopenia and outcomes by treatment group and enrollment era, we fitted a multivariable model including main effects for sarcopenia, treatment group, and enrollment era, covariates, all lower‐order interaction terms and a three‐way interaction term (sarcopenia × treatment group × enrollment era). Sarcopenia was defined using Martin's definition based on CT‐derived L3 skeletal muscle index (J Clin Oncol. 2013; 31:1539–1547. doi:10.1200/JCO.2012.45.2722).

## 4 Discussion

In this study, we investigated the predictive value of muscle mass in advanced NSCLC patients receiving systemic treatment with platinum‐doublet chemotherapy with or without a PD‐(L)1 inhibitor. Sarcopenia was present in 49.5% of patients with advanced NSCLC treated with first‐line systemic treatment at baseline. We identified that the presence of sarcopenia specifically predicts inferior treatment outcomes in patients treated with CITx both in terms of PFS and OS, which were not recapitulated in those treated with CTx only. Collectively, we were able to provide evidence for the predictive value of sarcopenia in treatment outcomes in patients with advanced NSCLC in the context of different systemic treatment modalities.

The emergence of immunotherapy vastly improved treatment methods and prognosis; the KEYNOTE‐042 study showed that patients with PD‐L1 TPS ≥ 50% had prolonged survival when treated with pembrolizumab alone compared to platinum‐based chemotherapy [21]. This result proved that PD‐L1 expression successfully predicts better outcome in NSCLC patients with PD‐L1 TPS ≥ 50% when given immunotherapy compared to platinum‐doublet chemotherapy. Studies such as KEYNOTE‐189 [10] and KEYNOTE‐407 [11] further advanced treatment paradigm by demonstrating improved OS and PFS in advanced NSCLC patients receiving PD‐(L)1 blockade plus platinum‐based chemotherapy compared to those receiving just platinum‐based chemotherapy, irrespective of PD‐L1 expression. Based on these studies, the current first‐line standard treatment for advanced NSCLC without druggable genomic alterations is a combination of PD‐(L)1 blockade and platinum‐based chemotherapy [13]. Nonetheless, there was no thoroughly validated predictive biomarker for the efficacy of PD‐(L)1 blockade plus platinum‐based chemotherapy. Markers such as PD‐L1 expression, tumour mutation burden and specific genomic alteration have already been explored and proven unhelpful [22]. Although sarcopenia has been linked to outcomes and toxicity in NSCLC patients treated with immune checkpoint inhibitors [14, 15, 16, 23], it remained unclear whether these associations are predictive of treatment response or merely prognostic. Therefore, we analysed treatment outcomes of patients treated with CITx or CTx based on the presence of sarcopenia and confirmed the predictive ability of sarcopenia. To our knowledge, this study is the first to assess the specific association between sarcopenia and treatment outcomes by comparing outcomes between patients treated with PD‐(L)1 inhibitors plus chemotherapy and those treated with just platinum‐doublet chemotherapy.

Patients with advanced cancer often carry metabolic and immunologic derangements, notably chronic systemic inflammation, leading to sarcopenia and adipose tissue wasting. Our study demonstrated that the quantity of skeletal muscle was an independent predictor of inferior CITx outcomes, which may be explained by the following biological mechanisms. As skeletal muscle cells serve endocrinologic functions by secreting myokines that regulate and modulate immune response, loss of skeletal muscle mass could contribute to diminished anti‐tumour immune response mediated by checkpoint blockades [15]. Cancer‐induced chronic inflammation itself releases pro‐inflammatory cytokines, leading to muscle breakdown. This catabolic state is thought to promote angiogenesis and immunosuppression, further dampening immunotherapy response [24]. We acknowledge that the observed effect sizes were modest. However, given the high prevalence and lethality of advanced lung cancer, even modest relative differences may translate into meaningful clinical impact at the population level. Unlike established biomarkers such as PD‐L1 expression, sarcopenia identified subgroups in which treatment benefit differed, suggesting that its predictive value may be particularly relevant in high‐risk patients with low muscle mass. Beyond its role as a predictive biomarker, sarcopenia represents a potentially modifiable condition. Randomized trials such as CHALLENGE have demonstrated the feasibility and survival benefit of exercise‐based interventions in colorectal cancer [25], supporting the translational potential of similar strategies in NSCLC. Future prospective trials are warranted to determine whether targeted nutritional and exercise‐based interventions can mitigate sarcopenia and enhance treatment benefit, particularly in high‐risk, sarcopenia‐positive subgroups [25, 26].

Based on the results of our study, several future studies can be conceived. First, prospective intervention studies using sarcopenia as a stratification factor can be designed to track the clinical relevance of sarcopenia on the treatment outcomes. Second, mechanistic studies elucidating the impact of sarcopenia on the tumour immune microenvironment, particularly its interaction with immune cell infiltration or cytokine signalling pathways, may provide insights into the biological underpinnings of treatment resistance in sarcopenic patients receiving checkpoint blockade therapy. Third, whether interventions focused on sarcopenia specifically and significantly improve response to systemic treatment including checkpoint blockade would be another intriguing area of investigation. For example, serial tracking of dynamic changes in body composition and inflammatory cytokine levels can be conducted to evaluate whether maintenance or enhancement of muscle mass through physical exercise can significantly impact not just metabolic conditions but treatment outcomes. Additionally, further exploration on the pharmacologic intervention for key mediators of sarcopenia or cachexia can be designed to explore whether such intervention can bring therapeutic benefit in patients with sarcopenia. Currently, novel drugs such as ponsegromab can be utilized in patients with cancer‐related cachexia [27]. Whether targeting sarcopenia and other metabolic dysregulations in patients with cancer would be a viable strategy to enhance treatment efficacy and improve overall clinical outcomes remains an important question for future investigation.

Our study has several limitations. Because treatment assignment was not randomized, our results are susceptible to confounding by indication; despite multivariable adjustment, residual confounding from unmeasured factors may partly explain differences between treatment groups. Given the long enrollment period, temporal bias related to evolving supportive care is a potential concern; however, adjustment for enrollment era and the absence of significant era‐related interaction terms suggest that differential benefit from chemoimmunotherapy versus chemotherapy was consistent across study periods. Because this was a single‐centre, single‐department retrospective study conducted in a Korean population, the generalizability of our findings to other ethnic groups, institutions and healthcare systems may be limited. External validation in diverse, multicentre cohorts is warranted. Although the Martin definition of sarcopenia was used as the primary classification due to its widespread use in prior studies, it was originally derived from Western populations and may not fully capture ethnic variability in body composition. Notably, sarcopenia defined using Korean population‐specific thresholds was entirely encompassed within the Martin‐defined sarcopenia group (Figure S3), showing good agreement. While our findings were robust across definitions, the absence of validated Asian‐specific cut‐offs remains a limitation that warrants further investigation. Lifestyle variables such as dietary habits, physical activity levels, and alcohol consumption, and functional data, including cardiopulmonary exercise test or handgrip strength, were not available. Future prospective studies incorporating detailed lifestyle assessments and functional status are warranted. Patients treated with immunotherapy monotherapy were excluded due to limited use and lack of insurance coverage during the study period; this should be considered when interpreting the results. Cachexia, which may overlap with sarcopenia and independently affect outcomes, could not be assessed in this cohort due to the lack of comprehensive nutritional and weight‐loss data [28]. Future studies incorporating standardized cachexia definitions and longitudinal nutritional assessments will be of interest. Currently, only a minority of patients receive chemotherapy alone in the first‐line metastatic setting, typically due to contraindications or limited access to immunotherapy. However, our findings may be transferable to later lines of therapy, where chemotherapy without immunotherapy remains more commonly used. The finding that sarcopenia has limited predictive value in patients not treated with immunotherapy is consistent with evidence reported in prostate cancer, which merits further investigation [29].

In conclusion, our study demonstrates the predictive value of sarcopenia for treatment outcomes in patients with advanced NSCLC receiving systemic therapy. Sarcopenia was associated with significantly worse progression‐free and OS among patients treated with chemoimmunotherapy, but not among those treated with chemotherapy alone, suggesting a treatment‐specific effect modification. Elucidating the biological mechanisms underlying this differential association represents an important area for future translational research. Prospective studies are warranted to determine whether early identification and targeted interventions for sarcopenia such as nutritional support and structured exercise programmes aimed at improving muscle mass, metabolic health and immunocompetence can translate into improved outcomes in patients receiving chemoimmunotherapy [25, 26]. Such an integrated approach may ultimately contribute to the development of novel supportive and adjunctive strategies for patients with advanced NSCLC.

## Funding

This work was supported by funding from the Ministry of Science and ICT of the Republic of Korea (RS‐2023‐00261820 to S.K.; RS‐2023‐00231864 to N.H.; RS‐2024‐00348654 to C.G.K.; RS‐2025‐02214844 to H.R.K.; RS‐2025‐18362970 to H.R.K.), Korea Health Industry Development Institute (KHIDI) funded by the Ministry of Health & Welfare, Republic of Korea (grant number: HI22C0452 to N.H.; RS‐2022‐KH126457 to N.H.), Severance Hospital Research fund for clinical excellence (C‐2023‐0040 to C.G.K.), Ministry of Trade Industry and Energy of the Republic of Korea (NRF‐20022947 to H.R.K.), Young Medical Scientist Research Grant through the Seokchunnanum Foundation (SCY2507 to N.H.), and National Research Foundation of Republic of Korea (NRF‐2021R1A2C2094629 to H.R.K.; NRF‐2022R1C1C1006807 to N.H).

## Ethics Statement

The Institutional Review Board of Yonsei University College of Medicine approved this study (IRB approved no. 4‐2023‐0034).

## Consent

The authors have nothing to report.

## Conflicts of Interest

Chang Gon Kim reported consulting or advisory role from Pfizer, Amgen, Novartis, Johnson & Johnson/Janssen and Ono Pharmaceutical, speakers' bureau from Takeda, Boehringer Ingelheim, Novartis, Merck, AstraZeneca, MSD Oncology, Hanmi and Dong‐A ST, and research funding from Ipsen. Hye Ryun Kim reported consulting or advisory role from Bayer, AstraZeneca, Bristol Myers Squibb, Takeda and Yuhan, speakers' bureau from AstraZeneca, Bristol Myers Squibb and Genentech/Roche, and research funding from Yuhan, Takeda, AstraZeneca, AFFIMED and Maia Biotechnology. Others have declared no conflicts of interest.

## Supporting information


**Table S1:** Treatment regimen according to sarcopenia status.
**Table S2:** Association of sarcopenia and treatment groups with progression‐free survival and overall survival in study patients.
**Table S3:** Hazard ratios of sarcopenia for progression‐free survival and overall survival by treatment groups using various sarcopenia definitions as sensitivity analyses.
**Table S4:** Association of L3 skeletal muscle, subcutaneous fat and visceral fat index with progression‐free survival and overall survival.
**Table S5:** Association of combined sarcopenia and treatment groups with outcomes.
**Figure S1:** Examples of body composition assessment at third lumbar spine level (L3) from non‐contrast computed tomography scan images.
**Figure S2:** Kaplan–Meier survival curves by treatment groups (CTx, platinum‐based chemotherapy alone; CITx, PD‐(L)1 immunotherapy combined with platinum‐based chemotherapy) for (A) progression‐free survival and (B) overall survival.
**Figure S3:** Agreement between three sarcopenia definitions. Orange circle: Martin's definition^1^; blue triangle: sex‐specific median threshold; Korean‐specific definition: threshold derived from Korean healthy population^2^.

## Data Availability

Data are available upon reasonable request.
