# Supplementary material for: Sarcopenia Predicts Outcome After Chemoimmunotherapy, Not Chemotherapy, in Advanced Lung Cancer: Single‐Centre Retrospective Study
Source: J Cachexia Sarcopenia Muscle. 2026 Mar 25;17(2):e70243. doi: 10.1002/jcsm.70243 (PMC13140918; doi:10.1002/jcsm.70243)
Supplement: Supplementary file 1 — Table S1: Treatment regimen according to sarcopenia status. Table S2: Association of sarcopenia and treatment groups with progression‐free survival and overall survival in study patients. Table S3: Hazard ratios of sarcopenia for progression‐free survival and overall survival by treatment groups using various sarcopenia definitions as sensitivity analyses. Table S4: Association of L3 skeletal muscle, subcutaneous fat and visceral fat index with progression‐free survival and overall survival. Table S5: Association of combined sarcopenia and treatment groups with outcomes. Figure S1: Examples of body composition assessment at third lumbar spine level (L3) from non‐contrast computed tomography scan images. Figure S2: Kaplan–Meier survival curves by treatment groups (CTx, platinum‐based chemotherapy alone; CITx, PD‐(L)1 immunotherapy combined with platinum‐based chemotherapy) for (A) progression‐free survival and (B) overall survival. Figure S3: Agreement between three sarcopenia definitions. Orange circle: Martin's definition1; blue triangle: sex‐specific median threshold; Korean‐specific definition: threshold derived from Korean healthy population2. [file JCSM-17-e70243-s001.pdf]

Supplementary materials

Sarcopenia Predicts Outcome After Chemoimmunotherapy, Not Chemotherapy, in Advanced Lung Cancer: Single-Center Retrospective Study

Hyojin Lee[1*], Chang Gon Kim[1*], Sookyeong Han[2,3], Su Kyoung Park[4], Hong In Yoon[5], Kyung Hwan Kim[5], Hyo Sup Shim[6], Min Hee Hong[1], Ja Hyun Yeo[1], Sangwoo Kim[7], Sang Hyun Hwang[8], Hye Ryun Kim[1,**], Namki Hong[2,3,**]

[1]Division of Medical Oncology, Department of Internal Medicine, Yonsei Cancer Center, Yonsei University College of Medicine, Seoul, Republic of Korea
[2]Department of Internal Medicine, Endocrine Research Institute, Yonsei University College of Medicine, Seoul, Republic of Korea
[3]Institute for Innovation in Digital Healthcare (IIDH), Yonsei University Health System, Seoul, Republic of Korea
[4]Department of Medical Records, Severance Hospital, Yonsei University College of Medicine, Seoul, Republic of Korea
[5]Department of Radiation Oncology, Yonsei Cancer Center, Yonsei University College of Medicine, Seoul, Republic of Korea
[6]Department of Pathology, Yonsei University College of Medicine, Seoul, Republic of Korea
[7]Department of Biomedical Systems Informatics and Brain Korea 21 PLUS Project for Medical Science, Yonsei University College of Medicine, Seoul, Republic of Korea
[8]Department of Nuclear Medicine, Severance Hospital, Yonsei University College of Medicine, Seoul, Republic of Korea

*HL and CGK contributed equally.
**HRK and NH contributed equally.

Correspondence to Hye Ryun Kim; nobelg@yuhs.ac (+82-2-2228-1932) and Namki Hong; nkhong84@yuhs.ac (+82-2-2228-0883)

Supplementary Table 1. Treatment regimen according to sarcopenia status

Treatment regimens	Overall (n, %)	With sarcopenia (n, %)	Without sarcopenia (n, %)	P-value
Pembrolizumab/Pemetrexed/Carboplatin	387 (33.0)	193 (33.2)	194 (32.7)	0.515
Pemetrexed/Cisplatin	285 (28.3)	147 (25.3)	138 (23.3)	
Paclitaxel/Carboplatin	174 (14.7)	78 (13.4)	96 (16.2)	
Pembrolizumab/Paclitaxel/Carboplatin	146 (12.4)	72 (12.4)	74 (12.5)	
Gemcitabine/Carboplatin	100 (8.5)	53 (9.1)	47 (7.9)	
Pemetrexed/Carboplatin	61 (5.2)	26 (4.5)	35 (5.9)	
Pembrolizumab/Pemetrexed/Cisplatin	7 (0.6)	5 (0.9)	2 (0.3)	
Nivolumab/Paclitaxel/Carboplatin	5 (0.4)	4 (0.7)	1 (0.2)	
Tislelizumab/Pemetrexed/Carboplatin	3 (0.3)	1 (0.2)	2 (0.3)	
Atezolizumab/Paclitaxel/Carboplatin	2 (0.2)	1 (0.2)	1 (0.2)	
Durvalumab/Gemcitabine/Carboplatin	2 (0.2)	0 (0.0)	2 (0.3)	
Docetaxel/Carboplatin	1 (0.1)	1 (0.2)	0 (0.0)	
Durvalumab/Pemetrexed/Carboplatin	1 (0.1)	0 (0.0)	1 (0.2)	

Values are presented as number (column percentage).

Supplementary Table 2. Association of sarcopenia and treatment groups with progression-free survival and overall survival in study patients

Predictors	Univariate model	
	Unadjusted hazard ratio (95% CI)	P-value
Outcome: PFS		
Sarcopenia	1.17 (1.04 to 1.31)	0.010
CITx (ref: CTx)	0.69 (0.61 to 0.77)	<0.001
Outcome: OS		
Sarcopenia	1.22 (1.08 to 1.39)	0.002
CITx (ref: CTx)	0.87 (0.76 to 0.98)	0.029

Abbreviations: ref, reference group.

Supplementary Table 3. Hazard ratios of sarcopenia for progression-free survival and overall survival by treatment groups using various sarcopenia definitions as sensitivity analyses

			Univariate model		Multivariable model*		
Outcomes	Treatment groups	Predictor	Unadjusted HR (95% CI)	P-value	Adjusted HR (95% CI)	P-value	P for interaction† (sarcopenia × treatment group)
Median definition (sex-stratified median of L3 skeletal muscle index)							
PFS	CITx	Sarcopenia	1.34 (1.12 to 1.60)	0.001	1.31 (1.05 to 1.64)	0.017	0.005
	CTx		0.98 (0.83 to 1.15)	0.840	0.86 (0.70 to 1.06)	0.171	
OS	CITx	Sarcopenia	1.35 (1.11 to 1.62)	0.002	1.42 (1.12 to 1.80)	0.003	0.009
	CTx		1.03 (0.86 to 1.21)	0.771	0.87 (0.70 to 1.07)	0.190	
Korean-specific definition (L3 skeletal muscle index threshold derived from Korean population)							
PFS	CITx	Sarcopenia	1.40 (1.12 to 1.75)	0.003	1.30 (1.01 to 1.69)	0.049	0.033
	CTx		1.12 (0.92 to 1.36)	0.228	0.93 (0.74 to 1.17)	0.528	
OS	CITx	Sarcopenia	1.63 (1.29 to 2.06)	<0.001	1.58 (1.20 to 2.08)	0.001	0.015
	CTx		1.21 (0.99 to 1.48)	0.057	1.02 (0.80 to 1.29)	0.589	

*Variables included in the multivariable Cox proportional hazard model: sarcopenia, treatment groups (CITx vs. CTx), enrollment era (defined according to whether patients were enrolled before or after the introduction of reimbursement by national health insurance), age, sex, body mass index, smoking history (pack-years), neutrophil-to-lymphocyte ratio, lung immune prognostic index (LIPI) score, pathology type, performance status, and PD-L1 expression. † To evaluate potential effect modification of the association between sarcopenia and outcomes by treatment group and enrollment era, we fitted a multivariable model including main effects for sarcopenia, treatment group, and enrollment era, covariates, all lower-order interaction terms, and a three-way interaction term (sarcopenia × treatment group × enrollment era). Abbreviations: ref, reference group; PFS, progression-free survival; OS, overall survival; HR, hazard ratio; CI, confidence interval.

Supplementary Table 4. Association of L3 skeletal muscle, subcutaneous fat, and visceral fat index with progression-free survival and overall survival

Outcomes	Treatment groups	Predictor (per 1 SD increment)	Adjusted HR (95% CI)*	P-value
PFS	CITx	Skeletal muscle index	0.89 (0.80 to 0.99)	0.049
		Visceral fat index	1.04 (0.93 to 1.18)	0.444
		Subcutaneous fat index	0.99 (0.88 to 1.12)	0.933
	CTx	Skeletal muscle index	0.98 (0.89 to 1.08)	0.686
		Visceral fat index	1.04 (0.94 to 1.15)	0.462
		Subcutaneous fat index	1.05 (0.94 to 1.16)	0.404
OS	CITx	Skeletal muscle index	0.83 (0.74 to 0.93)	0.002
		Visceral fat index	1.08 (0.95 to 1.24)	0.214
		Subcutaneous fat index	1.03 (0.90 to 1.18)	0.694
	CTx	Skeletal muscle index	0.99 (0.89 to 1.09)	0.882
		Visceral fat index	0.95 (0.85 to 1.07)	0.440
		Subcutaneous fat index	1.07 (0.95 to 1.20)	0.275

*Variables included in the multivariable Cox proportional hazard model: skeletal muscle index (standardized), visceral fat index (standardized), subcutaneous fat index (standardized), age, sex, enrollment era (defined according to whether patients were enrolled before or after the introduction of reimbursement by national health insurance), body mass index, smoking history (pack-years), neutrophil-to-lymphocyte ratio, lung immune prognostic index (LIPI) score, pathology type, performance status, and PD-L1 expression.

Supplementary Table 5. Association of combined sarcopenia and treatment groups with outcomes

Outcomes	Predictors	Unadjusted HR (95% CI)	P-value	Adjusted HR (95% CI)*	P-value
PFS	CTx and sarcopenia	1.00 (referent)		1.00 (referent)	
	CTx and no sarcopenia	0.89 (0.75 to 1.04)	0.141	0.97 (0.82 to 1.15)	0.768
	CITx and sarcopenia	0.72 (0.61 to 0.85)	<0.001	0.67 (0.55 to 0.81)	<0.001
	CITx and no sarcopenia	0.59 (0.50 to 0.70)	<0.001	0.54 (0.44 to 0.65)	<0.001
OS	CTx and sarcopenia	1.00 (referent)		1.00 (referent)	
	CTx and no sarcopenia	0.88 (0.74 to 1.03)	0.117	1.01 (0.85 to 1.20)	0.876
	CITx and sarcopenia	0.94 (0.79 to 1.12)	0.463	0.96 (0.78 to 1.17)	0.697
	CITx and no sarcopenia	0.70 (0.59 to 0.84)	<0.001	0.70 (0.57 to 0.86)	0.001

*Variables included in the multivariable Cox proportional hazard model: sarcopenia, treatment groups (CITx vs. CTx), enrollment era (defined according to whether patients were enrolled before or after the introduction of reimbursement by national health insurance), age, sex, body mass index, smoking history (pack-years), neutrophil-to-lymphocyte ratio, lung immune prognostic index (LIPI) score, pathology type, performance status, and PD-L1 expression.

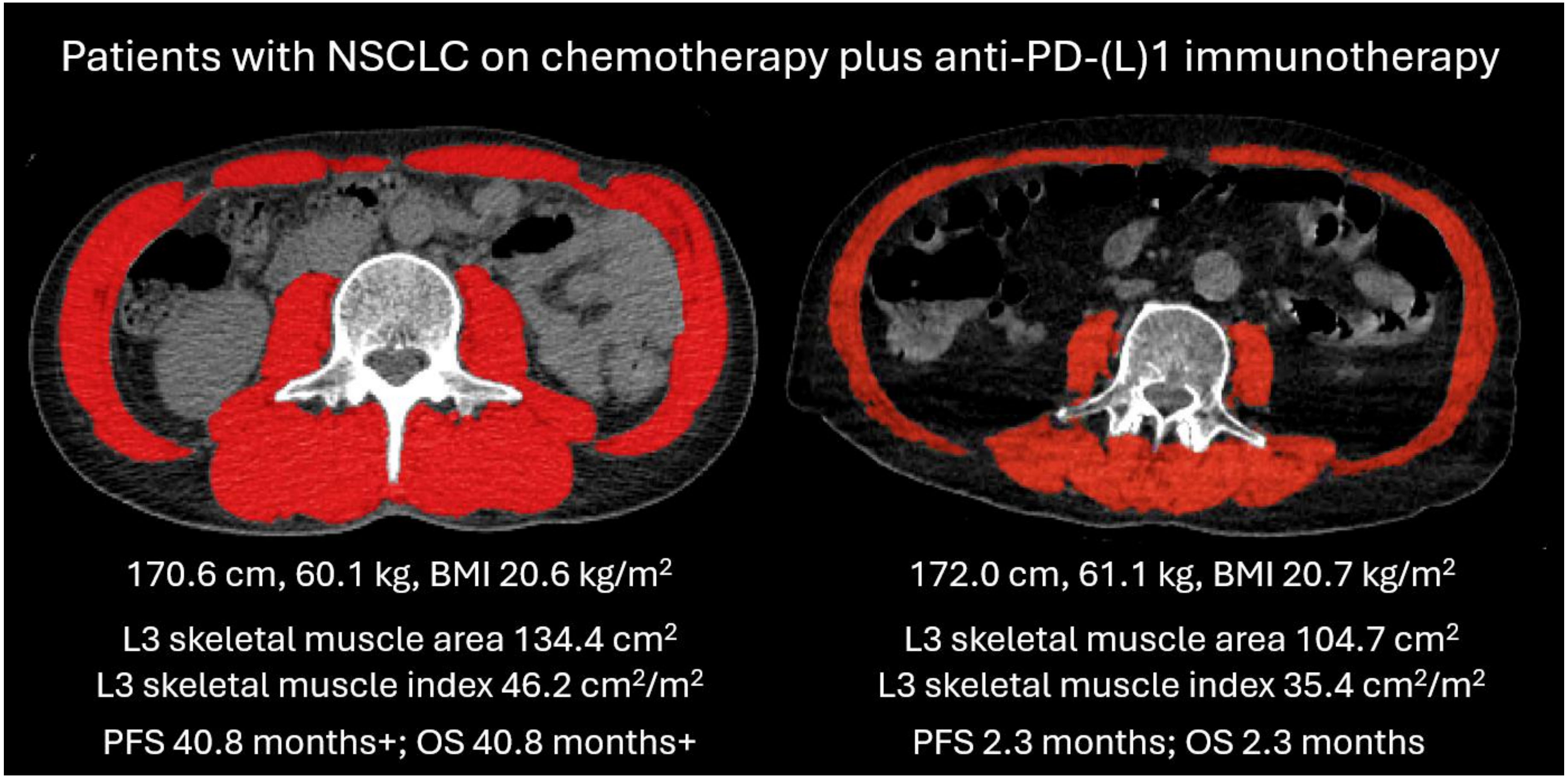

Supplementary Figure 1. Examples of body composition assessment at third lumbar spine level (L3) from non-contrast computed tomography scan images

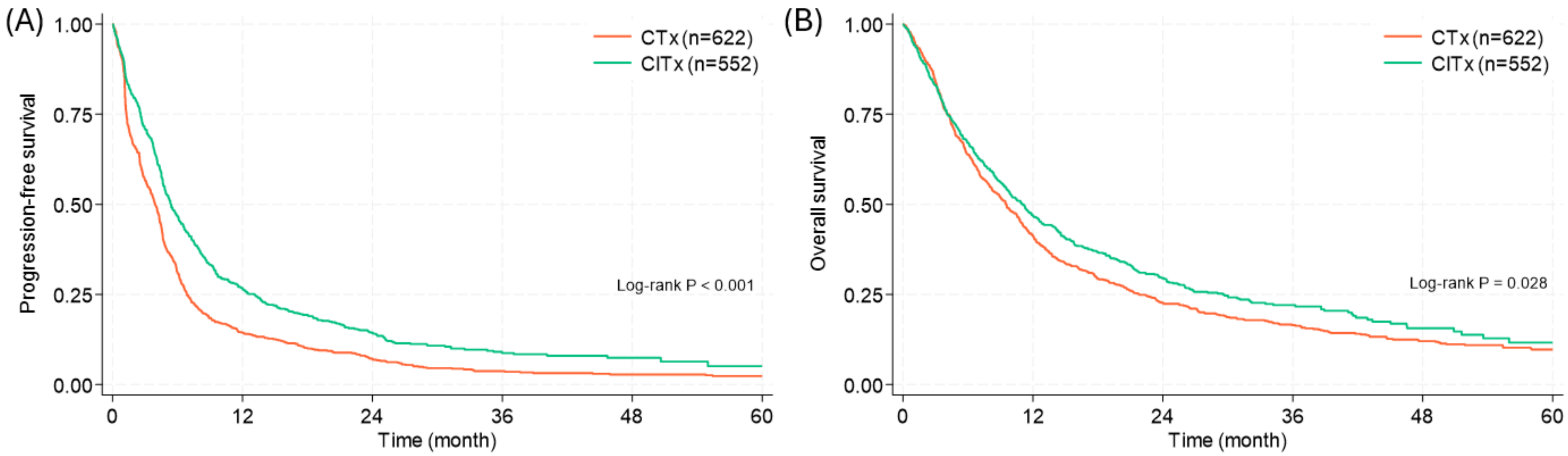

Supplementary Figure 2. Kaplan-Meier survival curves by treatment groups (CTx, platinum-based chemotherapy alone; CITx: PD-(L)1 immunotherapy combined with platinum-based chemotherapy) for (A) progression-free survival and (B) overall survival.

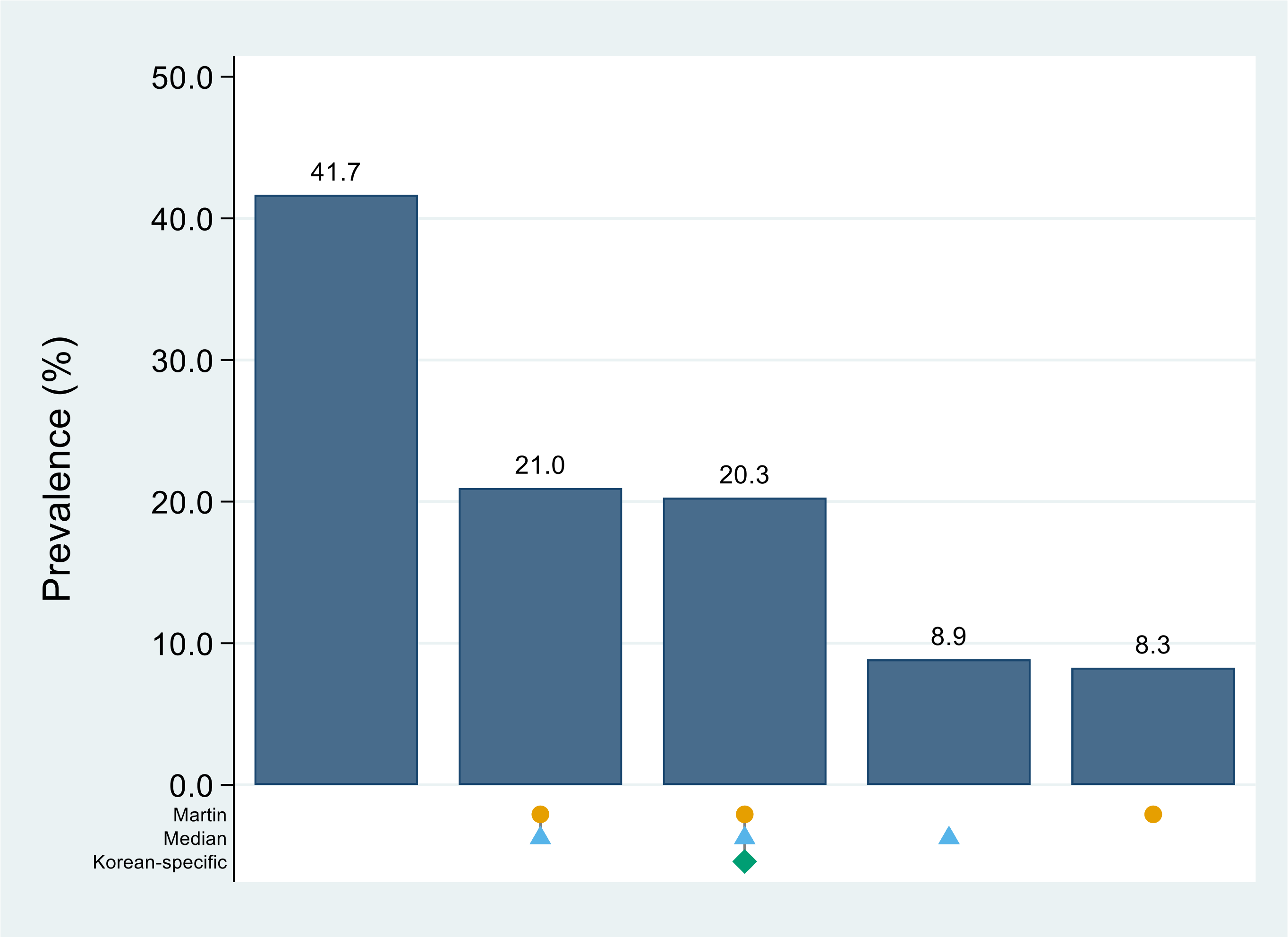

Supplementary Figure 3. Agreement between three sarcopenia definitions. Orange circle: Martin's definition[1]; blue triangle: sex-specific median threshold; Korean-specific definition: threshold derived from Korean healthy population[2]

References

1. Martin L, Birdsell L, Macdonald N, Reiman T, Clandinin MT, McCargar LJ, et al. Cancer cachexia in the age of obesity: skeletal muscle depletion is a powerful prognostic factor, independent of body mass index. *J Clin Oncol* 2013;**31**:1539-1547.

2. Yoon JK, Lee S, Kim KW, Lee JE, Hwang JA, Park T, et al. Reference Values for Skeletal Muscle Mass at the Third Lumbar Vertebral Level Measured by Computed Tomography in a Healthy Korean Population. *Endocrinol Metab* 2021;**36**:672-677. https://doi.org/10.3803/EnM.2021.1041

http://www.e-enm.org/journal/view.php?number=2174.
